# Regional Diffusion of Divorce in Turkey

**DOI:** 10.1007/s10680-017-9441-5

**Published:** 2017-10-03

**Authors:** Kim Caarls, Helga A. G. de Valk

**Affiliations:** Netherlands Interdisciplinary Demographic Institute (NIDI)/Koninklijke Nederlandse Akademie van Wetenschappen (KNAW)/University of Groningen (UG), Lange Houtstraat 19, 2511 CV The Hague, The Netherlands

**Keywords:** Divorce, Turkey, Regional context, Multilevel, Event history analysis

## Abstract

While demographic change has been well documented for many Western countries, much less is known about demographic transitions in other countries, including Turkey. Demographic change in European societies can be characterized by, amongst others, increased prevalence of divorce. Although it is often argued that life courses in Turkey follow a more traditional path, little is known on determinants and patterns of divorce, despite the major socioeconomic changes Turkey has undergone over the past decades. We study the levels of divorce of women in Turkey from 1973 to 2008 to explain patterns of divorce, looking at the role of individual characteristics and the regional context. We use the Demographic Health Surveys (2003/2008), complemented with regional data on divorce, urbanization, and GDP per capita. Applying a multilevel approach, distinguishing 12 regions, we hypothesize that regions where divorce is already more prevalent, more urbanized regions, and wealthier regions in terms of GDP per capita will increase the probability of divorce. Our analyses show that levels of divorce increased over the past decades but huge regional variation remains. Sociocultural and socioeconomic factors explain this trend, and in particular urbanization and GDP per capita are key determinants for divorce.

## Introduction

Divorce has been the topic of extensive research during the past decades (for overview articles see e.g., Amato 2010; Amato and James 2010; Härkönen 2013; Lyngstad and Jalovaara 2010; Wagner and Weiβ 2006). Increasing levels of divorce and other demographic changes have been linked to a shift in ideas toward family life where individualistic attitudes and higher acceptance of divergent family behaviours prevail (e.g. Härkönen 2013; Lesthaeghe 1995). While these processes have been well documented in Western countries, and in particular the USA and Europe, much less is known about demographic transitions in other regions of the world, including Turkey (Adams 2004; Rashad 2000; Tabutin and Schoumaker 2005).

It is often argued that life courses in Turkey still follow a more traditional path, yet we know little about family life transitions in general and the patterns and determinants of divorce in Turkey in particular. While higher divorce rates in Europe reflect, amongst others, the changed demographic behaviour (e.g. Härkönen 2013; Lesthaeghe 1983, 2010), it is often argued that the demographic transition in Turkey is not as advanced (Rashad 2000; Yüçeşahin and Özgür 2008). However, Turkey has undergone major socioeconomic changes over the past decades and this had its impact on family life transitions (Yüçeşahin and Özgür 2008; Yüksel-Kaptanoglu and Ergöçmen 2014). Turkey has not only witnessed a notable increase in divorce rates during the past decades (e.g. Demir 2013; Härkönen 2013; Kavas and Gündüz-Hoșgör 2010; Turkish Statistical Institute (TurkStat) 2011), this growth in levels of divorce corresponds to changes in Turkish family life in many domains during the last century: people marry later and have fewer children, and gender roles are said to be less traditional (Kavas and Thornton 2013).

These changes have been attributed to modernization processes, exposure to Western values, and socioeconomic changes. In addition, and reflecting these changes, there were several legislative developments in Turkey that affected the family. Regarding divorce, the two major changes were the introduction of the 1988 no-fault divorce law and the amendments to the Turkish Civil Code in 2001, both expanding women’s rights and advancing their position in the marriage (Arat 2010; Kavas and Gündüz-Hoșgör 2010; Yüksel-Kaptanoglu and Ergöçmen 2014).

Moreover, what also makes Turkey an interesting case is the huge regional variation: there are substantial differences between regions both in terms of economic development as well as in the spread of more modern values toward family life. Considering total fertility rates (TFR), for example, in some regions, these rates proximate those of European countries, while in other regions the TFR has remained high (Yüçeşahin and Özgür 2008; Yavuz 2008). While most studies on divorce concentrate on individual socioeconomic and demographic factors that predict whether a couple divorces or not (e.g. Heaton 2002; Wagner and Weiβ 2006), macro-level factors shape the context in which a couple’s union formation and dissolution takes place. Particularly, the role of regional variation within one country has remained relatively understudied (but see, e.g. Kalmijn and Unnk 2007; Lester 1999; Glass and Levchak 2014).

The aim of our paper is twofold. First, we examine divorce patterns in Turkey’s 12 regions over a 40-year time period, between 1967 and 2008. We study marriage cohort and period effects (e.g. the impact of different laws) amongst women aged 15–49 years. Second, we aim to pinpoint the mechanisms explaining the regional variation of divorce in Turkey, by investigating the importance of both macro- and micro-level predictors simultaneously. At the macro-level, both economic factors as well as the spread of modern values may influence the probability of divorce. More specifically, we investigate the role of regional variation on the probability of divorce by considering the average share of the regional gross domestic product (GDP) per capita within the total country-level GDP per capita, the level of population density, and crude divorce rates (CDR) for each of Turkey’s 12 regions. Data come from the Demographic Health Survey (DHS) (2003 and 2008 waves, http://www.dhsprogram.com) enriched with regional data from TurkStat. Multilevel discrete-time event history models are used to examine to what degree individual characteristics and the regional context influence divorce behaviour.

## Divorce in Turkey

In Western countries, divorce levels began rising sharply from the 1950 s onward (Lesthaeghe 2010). In Turkey, an increase in divorce rates occurred later (Härkönen 2013). Yet once divorce rates were rising, changes were substantial: while the CDR was only 0.27 in 1970, it increased to 1.40 in 2008 (TurkStat 2011). Parallel to this growth in levels of divorce are other changes in Turkish family life. In addition to a rising prevalence of divorce, fertility and mortality have been decreasing, bringing Turkey’s reproduction close to replacement level (DHS 2009). Furthermore, age at marriage increased, gender roles became more egalitarian, and the prevalence of patriarchal extended families declined (Yüksel-Kaptanoglu and Ergöçmen 2014). In the same vein, attitudes toward divorce became more tolerant (e.g. Kavas and Thornton 2013; TurkStat 2011). Although these changes have been connected to modernization processes and exposure to Western values, local values are not necessarily abandoned and modern and traditional values are simultaneously present in Turkish society (Kavas and Gündüz-Hoșgör 2010; Kavas and Thornton 2013; Yüçeşahin and Özgür 2008).

Although Turkish society can be characterized as patriarchal with low levels of gender equality (Göksel 2013; United Nations Development Programme (UNDP) 2005), progress has been made in this area (Yüksel-Kaptanoglu and Ergöçmen 2014). Women in Turkey increasingly challenge existing gender norms. For example, while financial decision-making was traditionally an exclusively male affair and joint bank accounts were basically non-existent, Turkish women today are managing their own bank accounts to a greater extent (Kavas and Gündüz-Hoșgör 2010). Despite these changes in gender relations that took place in Turkish family life, divorced women are still stigmatized and held accountable for their broken marriage (Kavas and Gündüz-Hoșgör 2011; Özar and Yakut-Cakar 2013).

With respect to divorce, there have been several relevant changes in Turkish law. Shortly after the foundation of the Turkish Republic in 1923, the Turkish Civil Code was adopted in 1926. This code provided women with more progressive rights compared to the Sharia law that was practiced before, such as equal inheritance and divorce rights for men and women. Although this code provided more gender equality, it was not until the 1980s that critiques were voiced against this code, which still reflected strong patriarchal notions regarding family matters (Arat 2010). In particular, articles underpinning women’s subordinate position were criticized, such as those defining the husband as head of the family and his wife as his helper and those formalizing separate ownership in marriage. These articles were especially detrimental for women during divorce, as they were consequently left without income or property.

Due to the efforts of the women’s movement (amongst others), some minor reforms took place before 2001. Turkey has signed several international conventions, such as the Convention on the Elimination of All Forms of Discrimination against Women monitored by the Committee on the Elimination of Discrimination against Women (Yüksel-Kaptanoglu and Ergöçmen 2014). Most notably, the no-fault divorce was introduced in 1988 [Divorce Law (No. 3444)], allowing divorce by mutual consent (Kavas and Gündüz-Hoșgör 2010).

Yet the most significant changes took place with the 2001 amendments of the Turkish Civil Code, which significantly expanded women’s rights, in particular with respect to women’s position in the marriage: articles that declared the husband to be the head of the household and his wife as his helper were deleted, the minimum age of marriage was raised to 18 for both men and women (it used to be 17 and 15, respectively), there were major changes in the property regime, from one based on separate ownership to one based on the sharing of property. This implied that in the event of divorce, women could now claim a share of the property registered in their husbands name if the property was acquired during the marriage (see Arat 2010; Kavas and Gündüz-Hoșgör 2010). Furthermore, while fathers originally had the final say over child custody, the new code provided fathers and mothers with equal leverage.

These changes in legislation took place in a context of socioeconomic developments, such as industrialization, rapid urbanization, educational expansion [particularly for women, e.g. the share of women with secondary education has increased from 34% in 1997 to 64% in 2010 (UNDP 2013)], and increased participation of women in economic spheres. These developments had their repercussions on Turkish family life, but these developments have not been distributed equally across Turkey. Modern and traditional values are simultaneously present in Turkish society (Cindoglu et al. 2008; Kavas and Gündüz-Hoșgör 2010; Kavas and Thornton 2013), resulting in diverse family systems across regions. While family regulations changed, practices such as arranged marriages, consanguineous marriages, and religious marriages maintained, in some regions more than others (Kavas 2010; Saadat 2015).

## Regional Variation in Divorce

In 2002, Turkey was included in the Nomenclature of Territorial Units for Statistics (NUTS) within the framework of the EU harmonization process, and 12 distinct regions (NUTS I) were identified. To a large extent, the sociocultural, sociodemographic and socioeconomic differences within Turkey are mirrored in these 12 regions (DHS 2009). The regions in the Western part of Turkey, encompassing Istanbul and İzmir, are the most urbanized and industrialized. The regions in the South have several growing industrial centres, such as Adana, Mersin, and Antalya. The capital city, Ankara, lies in Central Turkey. Besides this metropolis, the regions in Central Turkey are moderately industrial. The Northern region has a fertile coastal line and a mountainous interior, mainly occupied by small-scale farmers. The Eastern regions are economically the least developed and can be characterized by a rugged landscape.

While Turkey’s CDR (the number of divorces per thousand population in a given year) has been relatively low in recent years, the prevalence of divorce differs greatly between the different regions. Whereas the overall divorce rate in 2008 was 1.40, it ranged from 0.48 in Southeast Anatolia to 1.88 in the Aegean region (TurkStat 2011), which for example equals the crude divorce rates of the Netherlands [1.9 in 2009 (Eurostat 2015)]. Several macro-level studies indicated a range of contextual factors that are correlated with the cross-national variation in divorce rates, such as the role of the normative context (Amato and Keith 1991; Lesthaeghe 1995; Wagner and Weiβ 2006; Wolfinger 1999), legislative changes toward more liberal divorce legislation (Gonzaléz and Viitanen 2009; Stevenson and Wolfers 2007; Wolfinger 1999), family policies (Engelhardt et al. 2002), and female labour market participation (Diekmann and Schmidheiny 2004; Kalmijn and Unnk 2007). While these studies typically analyse between-country variation, we are interested in variation between regions. These regions can be considered a relevant context as they provide local opportunity structures (e.g. degree of urbanization, socioeconomic situation) and cultural milieus (e.g. acceptance or prevalence of divorce) that can affect individual behaviour (Hank 2002).

We expect that not only socioeconomic features of these regions play a major role in the level of diffusion of divorce (over and beyond the role of individual socioeconomic and sociodemographic characteristics), but also that different sociocultural factors influence the prevalence of divorce. For example, social norms regarding the use and acceptance of birth control vary strongly in the different regions in Turkey (Yüçeşahin and Özgür 2008). Previous studies on fertility in Turkey demonstrated that these different reproductive behaviours of women could be explained by, amongst others, diffusion processes (Yavuz 2008; Yüçeşahin and Özgür 2008). In particular, the cultural isolation of the eastern regions has prevented the diffusion of new and innovative reproductive behaviour, resulting in high fertility rates in the eastern part contrary to declining fertility trends elsewhere in Turkey (Yüçeşahin and Özgür 2008).

In a similar vein, we expect social norms regarding divorce to differ between the 12 regions. Consequently, these different norms will result in different CDR in these regions, since a higher prevalence of divorce reflects a higher cultural acceptance of divorce (Härkönen 2013). It can be expected that higher acceptance will decrease the stigmatization of divorce, making divorce more accessible for women. We therefore hypothesize that in regions where divorce has been more prevalent in the past, women’s probability of divorce in later years will be higher compared to regions with lower levels of divorce, net of other regional-level and individual-level characteristics (*Hypothesis 1*).

According to the diffusion theory, new behaviours typically start in metropolitan areas, where the upper and middle classes take the lead (Liefbroer and Doureleijn 2006; Nazio and Blossfeld 2002; Reed et al. 1999; Rogers 1983; Strang and Meyer 1993; Strang and Tuma 1993; Yavuz 2008). The level of urbanization varies greatly between the 12 regions, and we expect the level of divorce to vary accordingly, with a higher probability of divorce for women that live in more urbanized regions compared to women that live in intermediate or rural regions (*Hypothesis 2*).

There is also huge regional variation in terms of socioeconomic development. Regions in the Western part have a much larger share of the national gross domestic product (GDP) per capita than the Northern or Eastern regions (DHS 2009). Existing micro-level studies on divorce show mixed findings when it comes to the effect of economic circumstances on the probability of divorce (Aytaç and Rankin 2009; Härkönen and Dronkers 2006; Jalovaara 2003; Lyngstad and Jalovaara 2010). Several scholars studying these micro-level effects argue that acquiring a higher income has a stabilizing effect on marriages (the “income effect”). However, there is ample evidence from Europe and North America that this “income effect” will be outweighed by the so-called “independence effect”, which refers to an increase in female participation on the labour market resulting in more instable marriages (Lyngstad and Jalovaara 2010). Considering the Turkish context, we expect that the “independence effect” will be minimal, as female labour market participation is extremely low (ILO 2016). Yet few studies have analysed the impact of macroeconomic circumstances on divorce (for exceptions see, e.g. Amato and Beattie 2011; Bremmer and Kesselring 2004; Schaller 2012), and even fewer looked at the effect of macroeconomic conditions on the probability of divorce on a micro-level (e.g. Fischer and Liefbroer 2006). As with micro-level studies, previous macro-level studies typically discuss two opposing hypotheses. On the one hand, it is suggested that economic hardship increases the chance of divorce, with worse economic circumstances leading to more divorce and better economic conditions resulting in less divorce. On the other hand, difficult economic conditions may make it challenging to cover the costs of divorce, making divorce less likely (Fischer and Liefbroer 2006). To account for the specific Turkish context, where the social and financial cost for divorce is high, we expect the second hypothesis to hold: bad economic conditions will lead to less divorce (Aytaç and Rankin 2009; Fischer and Liefbroer 2006; Kavas and Gündüz-Hoșgör 2010) (*Hypothesis 3*).

## Changes Over Time: Cohort and Period Effects

Divorces in Turkey have significantly increased over the past 40 years, with CDR rising from 0.27 in 1970 to 1.40 in 2008 (TurkStat 2011). In addition to examining the role of individual *and* regional characteristics, we are also interested in explaining this increase in divorce over time, and to investigate whether period or cohort effects drive this change. Marriage cohort effects relate to the timing of marriage and the conditions that were present at that time. Consequently, different marriage cohorts have different attitudes, resources and practices, and these differences affect divorce rates (Härkönen 2013; Lyngstad and Jalovaara 2010). Period effects, in turn, affect all married couples, regardless of when they were married. The impact of new laws, for example, can bring about such period effects (González and Viitanen 2009; Yüksel-Kaptanoglu and Ergöçmen 2014).

We explored both cohort and period effects to investigate whether the change in CDR over time is the result of the emancipating effect of the no-fault divorce law in 1988 or of the amendments in Turkey’s Civil Code in 2001 (period effects) or whether these changes took place due to the changing social context wherein marriages took place (cohort effects). If period effects are present, significant changes should be seen between the periods after the 1988 and 2001 reforms (*Hypothesis 4a*). If cohort effects explain the changes over time, we should see more gradual changes over the studied time span (*Hypothesis 4b*).

Previous studies also addressed the issue whether the predictors of divorce change over time (Bernardi and Martínez-Pastor 2011; De Graaf and Kalmijn 2006; Härkönen and Dronkers 2006; Härkönen 2013). In particular, previous studies have shown the effect of women’s education on divorce to change over time. According to the so-called Goode hypothesis, the society presents a normative context that shapes individual divorce behaviour; when, in a given context, divorce is a relatively rare and often stigmatized event, it takes more resources to dissolve a marriage (Goode 1962). This implies that when divorce is not so common, higher educated women are more likely to break up (De Graaf and Kalmijn 2006; Härkönen and Dronkers 2006; Bernardi and Martínez-Pastor 2011). Additionally, women with higher socioeconomic status will be most likely to be the early adapters or innovators of new behaviours, such as divorce (Blossfeld et al. 1995; Hoem 1997).

For the Turkish fertility transition, higher educated women speaking Turkish were identified as the pioneers (Yavuz 2008). A higher socioeconomic status also makes women less sensitive to social conformities, even in spite of a sociocultural context in Turkey that typically represents patriarchal norms and values (Kavas and Gündüz-Hoșgör 2011). However, according to Goode’s hypothesis, when divorce becomes more common, lower educated women will also experience divorce and eventually, the effect of education will be reversed, with lower educated being more likely to divorce (De Graaf and Kalmijn 2006). We therefore anticipate that women with a higher education will have a higher risk of divorce in the earlier years of our observation period, when divorce was relatively uncommon (*Hypothesis 5)*.

Additionally, we expect that the effect of women’s education on divorce will be shaped by the regional context. Similarly, we expect that in regions where divorce is a rare phenomenon, the probability of divorce is higher for women with higher education, relative to women with less education (*Hypothesis 6*). Additionally, in less metropolitan, rural areas, where divorce is uncommon and less accepted, the probability of divorce is higher for women with higher education compared to women with less education (*Hypothesis 7*). With respect to wealthier regions, we hypothesize that the relation between the economic context and the probability of divorce will be most pronounced amongst those with less education; that is, better socioeconomic circumstances stabilize marriages amongst those with less education and the divorce risk of higher educated women will increase relative to those of lower educated women (*Hypothesis 8*).

## Micro-level Indicators of Divorce

The main focus of our study is on regional variance in divorce and changes over time. In order to do so we, however, control for a range of micro-level characteristics. We include indicators that have proved to be strong predictors of divorce in a range of earlier studies (for review articles, see Amato 2000, 2010; Amato and James 2010; Lyngstad and Jalovaara 2010; Härkönen 2013). In line with these previous works, we control for union (like the duration of marriage, age at marriage, age heterogamy, children born in the union, children from other than spouse) and individual characteristics (like childhood place of residence, mother’s literacy). In the Turkish context, there are several union-specific characteristics that also are essential to include (arranged marriages, consanguineous marriages) in addition to a distinction between the different ethnic groups in the country (indicated by mother tongue Kurdish) (Kavas 2010; Yavuz 2008). In line with the hypotheses formulated before, we are also particularly interested in the interaction between individual educational attainment (micro) and the regional diversity and cohort and period changes (macro). Educational attainment of the women is a key micro-level variable.

## Data and Method

The data used in our analyses are the Turkish Demographic and Health Survey (DHS), waves 2003 and 2008. In these surveys, households were randomly sampled within the 12 regions of Turkey. In each of these households, all women that were present in the household, or who usually live in that household, have been interviewed if they were between the ages of 15 and 49 and had been ever married. For more details about the DHS surveys, their target population and response rates, please see the full DHS 2003 and DHS 2008 reports (DHS 2004: p. 183, 2009: p. 222).

Two waves of data collection have been pooled, providing us with a robust number of divorced and married Turkish women (742 and 15,480, respectively),^1^ covering marriages that took place between 1967 and 2008. The survey contains a wide range of demographic and health-based questions, and it includes a history of women’s marriages. As the number of recorded marriages varies per wave, and the number of women with more than one marriage is limited, we will focus on first marriages only.^2^ We consider whether these first marriages ended in divorce or not.

Using the retrospective information, we constructed a person-period file with respondents’ information on a yearly basis. We followed respondents from the year of their first marriage until divorce or in case of censoring by the time of the survey or by the death of the spouse. Additionally, we excluded respondents from whom we did not have complete information concerning the start and end years of their marriage (*n* = 15 and *n* = 23, respectively). This resulted in a dataset consisting of 15,418 respondents; 726 respondents experienced divorce or separation. The first divorce occurred in 1973 and the last occurred in 2008. Our definition of divorce includes women who are living together in an unmarried or a married union as the Turkish DHS survey does not distinguish between them. Although we might therefore slightly overestimate the number of married women, we expect the extent of this bias to be minimal, as non-marital cohabitation hardly occurs in Turkey (Yavuz 2008).

Since we want to explore the effects of individual and context-level factors on women’s probability of divorce, we use multilevel discrete-time logistic regression models that enable us to simultaneously use explanatory variables at these two levels (i.e. individual and regional) (Snijders and Bosker 1999). The person-period file consisted of 222,616 person-years and we differentiated between 12 regions. We assessed the duration dependency by using the number of years of marriage. We tested for nonlinear effects, and the inclusion of a linear and a squared term fitted the data best. The time-varying variables were lagged with one year, which is in line with standard event history procedures (Singer and Willett 2003).

We first modelled a null model, which includes the random intercept and the variable for duration of marriage (cf. Hox 2002: p. 81). To account for the hierarchical structure of the data, all subsequent models include the random intercept. Next, we included all individual variables. In our subsequent models, we include the regional variables (due to our limited sample size at the regional level and to avoid multicollinearity, we decided to estimate our regional variables separately), and we additionally estimate models to examine period and marriage cohort effects. Finally, we investigate interaction effects to examine whether the effect of education has changed over time and whether the regional characteristics shape the effect of education.

### Measures of Contextual Variables

**Fig. 1:**
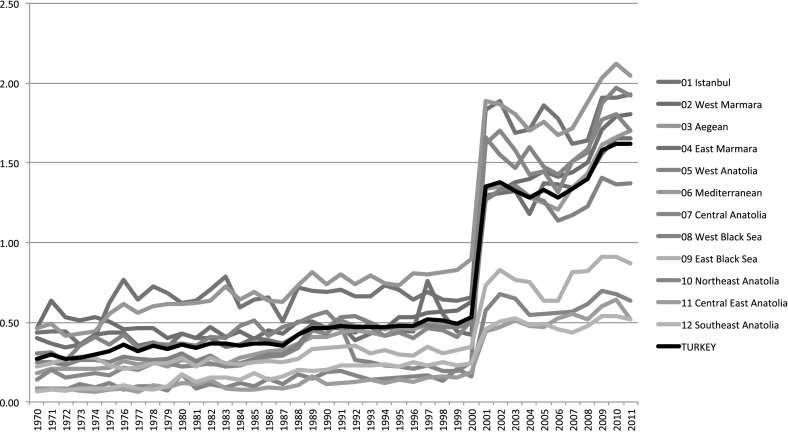
Crude divorce rates by province, 1970–2011 *Source*: Authors’ calculations (based on: Turkstat 2001–2009; SIS 1970–2000)

We distinguished 12 geographical regions (NUTS-1) as the region in which respondents living at the time of survey. Three variables were constructed on the regional level. First, we were interested in regional CDR. This information was not available for the entire time period we were interested in. We were, however, able to obtain information about divorces per province. Today, Turkey is divided in 81 provinces (before 1989, there were 67 provinces, but several changes between 1989 and 1999 resulted in 81 provinces since then [for detailed information about Turkey’s administrative divisions, see statoids.com/utr.html)]. Provincial crude divorce rates were available through marriage and divorce statistics from TurkStat [formally State Institute for Statistics (SIS)] for the period 2001–2008. For the period 1970–2000, the SIS provided only information about the total number of divorces per province. Using the six censuses that were carried out by the State Institute for Statistics from 1970–2000, we imputed the missing years to arrive at population estimates for each year, for each province. Crude divorce rates were then computed using the number of divorces and population estimates by province. Using these provincial crude divorce rates, we calculated the crude divorce rate for each of the 12 regions by taking the (weighted by population size) average divorce rate of the provinces in each region for each year (see Fig. 1, discussed below).

**Fig. 2:**
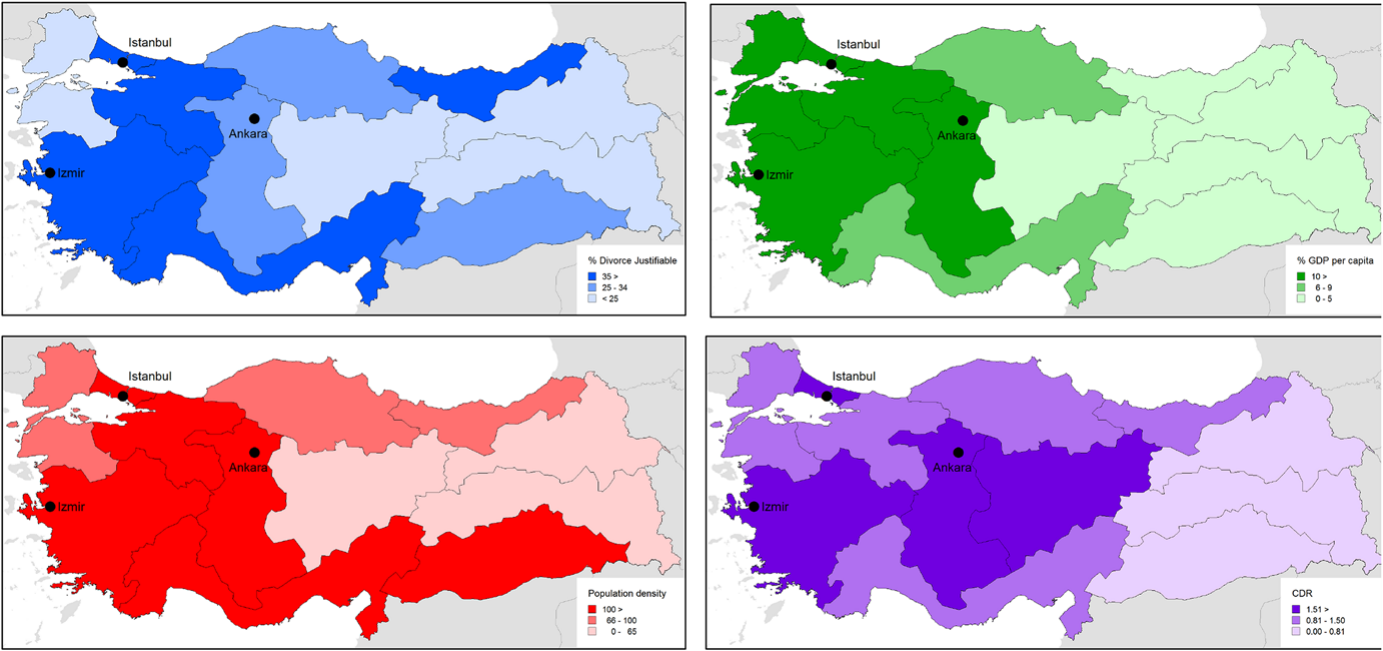
Maps of Turkey showing the regional distribution of “acceptance of divorce”, “share of GDP per capita”, “population density”, and “crude divorce rate (CDR)” (time of survey) *Source*: Authors’ calculations [based on: TurkStat 1987–2001 (GDP per capita); WVS 2007 (Acceptance of divorce); OECD 2015 (Population density); SIS 1970–2000 and TurkStat 2001–2009 (CDR)]

Second, using information about population density (population per km^2^) per region for the period 2001–2009 (OECD 2015) as a proxy for urbanization, we classified the 12 regions as rural (0–65), intermediate (66–99), and urban (100>). As this information was only available for a limited time period, we explored the variation over this distribution time, which appeared almost constant for all regions (see Fig. 2, discussed below). Therefore, we decided to include regional population density as a time-constant variable.

The third contextual variable is the average share of the regional GDP per capita within the total country-level GDP per capita for the period 1987–2000. Information concerning Turkish GDP on regional or provincial level was also only available from SIS for a limited period (1987–2001). Using population figures for each region, we first calculated regional GDP per capita (regional GDP/regional population) for the period 1987–2000. When examining the regional share of the total country-level GDP per capita for this time period, our analyses demonstrated little regional variation over time despite an overall increase in GDP (figures available upon request). This led us to include a time-constant variable that captures the average share of the regional GDP per capita as a percentage of the total country-level GDP per capita.

### Measures of Micro-variables

We controlled for a number of sociodemographic characteristics of the interviewed women. First, we controlled for the duration of the marriage. Women’s age at marriage was treated as a continuous variable, and a squared term was added to account for nonlinear effects. The educational level at time of survey was included, referring to the highest educational level attained, distinguishing between those who have had 0 = *no,* 1 = *primary,* 2 = *secondary,* and 3 = *tertiary education*. Ideally we would have included a time-varying measure of educational attainment; unfortunately, there was no information about the educational histories of the women available. However, since most women completed their education before getting marriage, we believe the bias to be minimal.

The same holds for respondent’s socioeconomic status. The DHS surveys have no variable capturing the respondent’s situation before marriage. We therefore included a variable indicating whether or not the respondent’s mother was literate, as a proxy of her socioeconomic status before marriage. Previous research also showed large differences in demographic behaviours between Kurdish and Turkish-speaking persons (Yavuz 2008; Yüçesahin and Özgür 2008). We thus distinguished between those with Kurdish and those with Turkish, Arabic or a different language as their native tongue. We included information about how the couples’ marriage was arranged: 0 = *by the couple themselves,* 1 = *by family*, and 2 = *other*. Additionally, since consanguineous marriages are relatively common in Turkey (Koç 2008), we controlled for this too.

Because the DHS surveys focus on women, the information about respondents’ partner is much less detailed. We did have information about the husband’s age at the time the union started, from which we constructed a categorical variable capturing the age difference between the spouses, with 0 = *wife older than husband*, 1 = *same age*, and 2 = *husband older than wife*.

Furthermore, we took respondents’ childhood residency (until age 12) into account, differentiating between respondents who 0 = *lived in a rural area*, or 1 = *lived in an urban area,* or 2 = *lived abroad*. Finally, the DHS surveys feature a fertility module, allowing us to find out exactly at what date women have had children. As the data also give us exact information on the start and end of the marriage, we were able to consider both whether or not the couple had children (time varying, lagged with one year) and whether the respondent had children that were born from someone other than the spouse.

To be able to examine cohort and period effects, we used various measures: marriage cohort was considered by using a continuous variable “year of marriage” (centred) and a categorical variable with 0 = *before 1980*, 1 = *1981*–*1990*, 2 = *1991*–*2000*, and 3 = *2001 and later.* Period was measured with a categorical variable, capturing the two legislative changes in 1988 and 2001, with 0 = *before 1988*, 1 = *1988*–*2000*, and 2 = *after 2001*. Table 1 provides the descriptive statistics of all variables used in the models (in person-years).

**Table 1:** Descriptive statistics of Turkish women aged 15–49 in person-years (*N* = 222,616)

	Mean (se)	%
Regional level (macro)		
Crude divorce rate (time varying, *t* − 1)	0.71 (0.00)	
GDP per capita (regional %)	0.10 (0.00)	
Population density		
Rural		12.7
Intermediate		34.6
Urban		52.7
Individual level (micro)		
Marriage duration	10.53 (0.02)	
Age at marriage	18.90 (0.01)	
Education		
None		19.05
Primary		58.33
Secondary		12.25
Tertiary		10.36
Literacy mother		
No		32.41
Yes		67.59
Children (time varying, *t* − 1)		
No		14.88
Yes		85.12
Children from other than spouse		
No		95.12
Yes		4.88
Age difference		
Wife older		7.67
Same age		7.52
Husband older		84.81
Mother tongue Kurdish		
No		86.11
Yes		13.89
Consanguinity		
No		74.72
Yes		25.28
Arranged marriage (no = ref.)		
Not arranged		33.8
Family		60.29
Other		5.91
Childhood residence		
Rural		54.91
Urban		43.78
Abroad		1.31
Year of marriage (continuous)	1986.17 (0.02)	
Marriage cohort		
Before 1980		29.21
1981–1990		39.98
1991–2000		25.88
2001 and later		4.93
Period: year		
Before 1988		16.82
1988–2000		50.83
2001 and later		32.35

*Source*: DHS (2003, 2008) (weighted means, standard errors, and percentages)

## Findings

### Regional Variation in Crude Divorce Rates and GDP per Capita

Our analyses show clear regional variation in CDR over time (Fig. 1). For the whole of Turkey, CDR rose markedly from 0.27 in 1970 to 1.40 in 2008. Although this upward trend is more or less visible for all regions, we observe large regional differences. Figures in Southeast Anatolia range from 0.06 in 1970 to 0.48 in 2008, reflecting the lowest CDR. Today, the highest CDR can be found in the Aegean region, where CDR ranged from 0.39 in 1970 to 1.88 in 2008. We also show how the difference in CDR between the 12 regions increased over time. While the variation in 1970 was 0.38, this increased to 1.40 in 2008.

Two notable increases in the CDR stand out: first, an increase after 1988, and second, a large jump in CDR as of 2001. Two law reforms could be responsible for these changes: the introduction of the 1988 no-fault divorce law, which enabled divorce by mutual consent, and the 2001 amendments of the Turkish Civil Code, which further improved women’s position, particularly in the event of divorce (Arat 2010; Kavas and Gündüz-Hoșgör 2010; Yüksel-Kaptanoglu and Ergöçmen 2014). The extent to which these period effects drive women’s probability of divorce will be discussed later.

In Fig. 2, we show four maps of Turkey, visualizing the regional distribution of the acceptance of divorce, the average share of the regional GDP per capita within the total country-level GDP per capita, population density, and CDR at the time of survey. Even though there is variation between the twelve regions in how these four variables are distributed, these maps demonstrate the notable differences between the West and East of Turkey, whereby the West is more favourable toward divorce, richer, more urban, and with higher CDR.

While the variation between regions changed considerably over time with respect to CDR, the average share of the regional GDP per capita within the total country-level GDP per capita did not change much over time. The Istanbul and East Marmara regions represent the wealthiest regions in terms of GDP per capita, with each on average 15% of the total GDP per capita. Next, the Aegean region and West Marmara are the wealthiest, encompassing on average 11% of Turkey’s GDP per capita. Northeast Anatolia, Central East Anatolia, and Southeast Anatolia are the poorest regions, representing on average 3, 4, and 5% of the national GDP per capita, respectively.

In the same vein, the distribution of the level of population density across the 12 regions is relatively stable. Only the Mediterranean region and Southeast Anatolia changed between 2001 and 2009, both from intermediate regions to urbanized regions. In 2008, half of the regions were urban (Istanbul, Aegean, East Marmara, West Anatolia, Mediterranean, and Southeast Anatolia). Three regions were intermediate (West Marama, West Black Sea, and East Black Sea), and three regions were rural (Central Anatolia, Northeast Anatolia, Central east Anatolia).

Finally, we additionally examined regional differences concerning the attitude towards divorce, since we argued that a higher prevalence of divorce reflects a higher cultural acceptance of divorce (Härkönen 2013). The acceptance towards divorce varies across the regions, ranging from very high acceptance in some regions (e.g. 53% in the Aegean region) to very low acceptance in other regions (e.g. 18% in West Marmara) (World Values Survey (WVS) 2007).^3^ Regional information concerning these attitudes was only available for 2007. Since we expect these attitudes to significantly vary over time (similar to regional CDR), we did not include this information in our multivariate analyses.

### Individual and Regional Determinants of Divorce in Turkey

We first fitted a null model, which includes the random intercept and the variable for duration of marriage (cf. Hox 2002: p. 81). Next, we include our micro-level control variables in Model 1, and in Models 2 we include our different macro-level variables. Due to the relatively small number of regions, we estimate separate models for each of our regional variables (Table 2 Models 2a, 2b, and 2c).

**Table 2:** Multilevel results predicting women’s divorce from regional and individual-level variables *Source*: DHS (2003, 2008)

	Model 0		Model 1		Model 2a		Model 2b		Model 2c	
	Coef.	(SE)	Coef.	(SE)	Coef.	(SE)	Coef.	(SE)	Coef.	(SE)
Individual level										
Marriage duration	−0.046***	0.006	0.012	0.008	0.004	0.009	0.012	0.008	0.012	0.008
Age at marriage			−0.171***	0.039	−0.175***	0.039	−0.177***	0.039	−0.176***	0.039
Age at marriage (sq)			0.004***	0.001	0.004***	0.001	0.004***	0.001	0.004***	0.001
Education (none = ref.)										
Primary			0.280**	0.140	0.278**	0.140	0.270**	0.140	0.265*	0.140
Secondary			0.733***	0.166	0.729***	0.166	0.719***	0.166	0.718***	0.166
Tertiary			0.695***	0.184	0.684***	0.184	0.676***	0.184	0.679***	0.184
Literacy mother			−0.130	0.092	−0.140	0.092	−0.123	0.092	−0.130	0.092
Children (time varying, * t* − 1)			−1.090***	0.099	−1.092***	0.099	−1.089***	0.099	−1.087***	0.099
Children from other than spouse			1.456***	0.115	1.454***	0.115	1.447***	0.115	1.452***	0.115
Age difference (wife older = ref.)										
Same age			−0.649***	0.174	−0.652***	0.174	−0.652***	0.174	−0.653***	0.175
Husband older			−0.833***	0.123	−0.836***	0.123	−0.835***	0.123	−0.837***	0.123
Mother tongue Kurdish			−0.472***	0.161	−0.447***	0.160	−0.436***	0.158	−0.426***	0.160
Consanguinity			−0.264***	0.107	−0.263***	0.106	−0.264***	0.106	−0.256**	0.107
Arranged marriage (no = ref.)										
Family			0.236***	0.093	0.240***	0.092	0.246***	0.093	0.249***	0.093
Other			0.845***	0.145	0.842***	0.145	0.841***	0.145	0.835***	0.145
Childhood residence (rural = ref.)										
Urban			0.477***	0.091	0.481***	0.091	0.475***	0.091	0.479***	0.091
Abroad			0.955***	0.230	0.964***	0.230	0.949***	0.230	0.937***	0.230
Marriage cohort (before 1980 = ref.)										
1981–1990			0.258**	0.122	0.210*	0.123	0.265**	0.121	0.266**	0.121
1991–2000			0.730***	0.135	0.617***	0.146	0.741***	0.134	0.743***	0.135
2001>			0.840***	0.189	0.609***	0.224	0.860***	0.188	0.860***	0.188
Regional level										
Crude divorce rate (time varying, *t* − 1)					0.212*	0.114				
Population density (rural = ref.)										
Intermediate							0.211*	0.132		
Urban							0.372***	0.136		
GDP per capita (regional %)									3.416***	1.438
Intercept	−5.327***	0.108	−3.825***	0.538	−3.767***	0.536	−3.975***	0.541	−4.064***	0.547
Random intercept	0.305***	0.078	0.164**	0.067	0.110	0.073	0.082	0.074	0.113*	0.061
Observations: *individual level*	222,616		205,994		205,910		205,994		205,994	
Observations: *regional level*	12		12		12		12		12	
Total explained variance^a^	0.033		0.174		0.178		0.180		0.180	
Unexplained variance: *regional level*	0.028		0.007		0.003		0.002		0.003	
Likelihood ratio test	33.960***		4.09**		1.03		0.46		1.67*	

^a^For details on the calculation of the total explained variance, see Snijders and Bosker (1999): 225

* *p* < .05; ** *p* < .01; *** *p* < .001

In Model 0, the random intercept exhibits significant variation, demonstrated by a likelihood ratio test comparing a multilevel model to an ordinary logistic regression *(p* = .000), and by the standard deviation of random intercepts (.305) being more than twice its standard error (.078). Although the intraclass correlation is not straightforwardly obtained in binomial models, we calculated the intraclass correlation in line with Snijders and Bosker (1999: p. 224). The intraclass correlation is 0.028, indicating a small but significant degree of dependence between the two levels. The between-region variance, i.e. the proportion of the total variance due to the variance between regions, which is 3% in the null model, is reduced to 1% in Model 1, after including the micro-level variables. This demonstrates that the between-region variance is largely related to the population composition in these regions, in as far as we accounted for that by our micro-level variables. The random intercept still reveals significant variation at the regional level. In Models 2, the between-region variation is further reduced to less than 1% Models 2 (0.3, 0.3, and 0.2%, respectively) and remains only significant in Model 2b. This indicates that the remaining variation between regions is mostly accounted for by the various regional characteristics.

Before coming to the core of our analyses, we briefly discuss the micro-level control variables introduced in Model 1. These variables are almost all significantly influencing the probability of divorce, in line with previous studies on the determinants of divorce. The exceptions are mother’s literacy and marriage duration, which were no longer significant after controlling for the other individual characteristics. The nonlinear effect of the age at marriage shows that marrying either very young or at late age increases the risk of divorce (see Lehrer (2008) for a similar finding in the USA). The effect of education on divorce is positive and significant in all models, conforming several previous studies (e.g. Blossfeld et al. 1995; Frank and Wildsmith 2005; Hall and Zhao 1995; Kalmijn et al. 2004). This means that higher educated Turkish women are more likely to divorce than their lower educated counterparts, net of the other individual characteristics we controlled for. Having children decreases the risk of divorce, except when these children are born from someone other than the spouse, in which case women have higher risks of divorce (Härkönen and Dronkers 2006).

Marriages where the husband is older are the most stable compared to marriages where the partners have the same age or when the wife is older than her husband (Gentleman and Park 1994; Janssen et al. 1999; Kalmijn and Poortman 2006). Marriages that are reflective of more patriarchal Turkish norms are less likely to dissolve, such as arranged (Jones 2007), consanguineous (Saadat 2015), and Kurdish marriages (Yavuz 2008). Finally, women that grew up in an urban setting or who have experienced living abroad are more likely to divorce compared to women who grew up in rural Turkey. Finally, controlling for marriage cohort, our findings illustrate that the more the recent marriage is, the more likely a divorce. These individual-level variables together explain about 17% of the total variation (see Snijders and Bosker 1999: p. 225 for the calculation of the explained variance in binomial multilevel models).

The effects of our control variables remain the same after including the regional-level explanatory variables: the time-varying indicator capturing the regional divorce rate (lagged 1 year) (Model 2a), the time-constant variable indicating the level of population density (Model 2b), and the time-constant variable referring to the average share of the regional GDP per capita within the total country-level GDP per capita (Model 2c). As expected, women’s probability of divorce is higher in regions where divorce has been more prevalent in the previous year (*Hypothesis 1*), and in regions that are more urbanized (*Hypothesis 2)*. These two hypotheses might provide tentative evidence for theories on the diffusion of innovations. Considering divorce as a “new behaviour”, we expected to find a higher risk of divorce in metropolitan areas (Nazio and Blossfeld 2002; Liefbroer and Doureleijn 2006; Rogers 1983; Strang and Meyer 1993; Strang and Tuma 1993; Yavuz 2008), and our analyses for Turkey confirm this. While previous evidence regarding the role of socioeconomic circumstances is mixed, we find that in wealthier and more developed regions, in terms of GDP per capita, the probability of divorce is higher (*Hypothesis 3*) (Aytaç and Rankin 2009; Fischer and Liefbroer 2006; Jalovaara 2003; Kavas and Gündüz-Hoșgör 2010; Lyngstad and Jalovaara 2010).

### Period and Cohort Effects on Divorce in Turkey

In Table 3, the results of our analyses on whether period or cohort effects shape women’s probability of divorce in Turkey are presented. Our previous models (Table 2), showed a strong positive effect of marriage cohort, indicating that those marrying later are more likely to divorce. In Models 1a–c (Table 3), we show again the effects of marriage cohort. In line with Fig. 1, which clearly demonstrated how the two liberating legislative changes in 1988, and most notably in 2001, shape the national and regional CDR in Turkey, we show the effect of period by including a set of dummy variables measuring the period before 1987, 1988–2000, and 2001 onwards (Models 2a–c). As the coefficients of our micro-level variables have not changed compared to the Models in Table 2, we show only the cohort, period, and regional variables here.

**Table 3:** Multilevel results predicting women’s divorce from regional- and individual-level variables: cohort and period effects

	Model 1a		Model 1b		Model 1c		Model 2a		Model 2b		Model 2c	
	Coef.	(SE)	Coef.	(SE)	Coef.	(SE)	Coef.	(SE)	Coef.	(SE)	Coef.	(SE)
Individual level^a^												
Year of marriage (≤1980 = ref.)												
1981–1990	0.210	0.123	0.265**	0.121	0.266**	0.121						
1991–2000	0.617***	0.146	0.741***	0.134	0.743***	0.135						
≥2001	0.609**	0.224	0.860***	0.188	0.860***	0.188						
Period (before 1988 = ref.)												
1988–2000							0.327***	0.129	0.348***	0.129	0.350***	0.129
2001>							0.630***	0.178	0.753***	0.141	0.755***	0.141
Regional level												
Crude Divorce rate (time varying, *t* − 1)	0.212*	0.114					0.145	0.145				
Population density (rural = ref.)												
Intermediate			0.211*	0.132					0.210	0.132		
Urban			0.372**	0.136					0.368***	0.137		
GDP per capita (regional %)					3.416**	1.438					3.37**	1.439
Intercept	−.3767***	0.536	−3.975***	0.541	−4.064***	0.550	−3.683***	0.541	−3.817***	0.545	−3.904***	0.550
Random intercept	0.110	0.536	0.082	0.074	0.113	0.061	0.125*	0.076	0.083	0.074	0.113*	0.062
Observations: *individual level*	205,910		205,994		205,994		205,910		205,994		205,994	
Observations: *regional level*	12		12		12		12		12		12	
Unexplained variance: *regional level*	0.003		0.002		0.003		0.004		0.002		0.003	
Total explained variance^b^	0.178		0.180		0.180		0.174		0.178		0.178	

^a^All models controlled for: marriage duration, age at marriage, age at marriage (squared), education, mother’s literacy, children (t − 1), children from other than spouse, age difference, mother tongue Kurdish, consanguinity, arranged marriage, childhood residence

^b^For details on the calculation of the total explained variance, see Snijders and Bosker (1999): 225

* *p* < .05; ** *p* < .01; *** *p* < .001

*Source*: DHS (2003, 2008)

Looking at the total variation explained, we see that the models including marriage cohort effects are slightly better in explaining women’s probability of divorce compared to the models with period effect. The models that include the categorical variable of marriage cohort have the highest variation explained, approximately 18% (Models 2a–c, Table 2). The risk of divorce for women married between 1991–2000 or 2001 and later is approximately two times higher than women married before 1980. Similarly, the risk of divorce in the period after 2001 is also two times higher for women compared to the risk of divorce in the period before 1988. This means that we find evidence of both period and cohort effects (*Hypotheses 4a* and *4b*).

### Individual- and Cross-Level Interactions

Table 4 shows the findings for the individual-level interactions to investigate whether the effect of education has changed over time. We do so by, using the continuous measure “year of marriage” (Models 1a–c), and cross-level interactions with the macro-variables (CDR, population density, and the average share of GDP per capita for each region) to see if they have a significant effect on the effect of education on divorce (Models 2a–c).

**Table 4:** Multilevel results predicting women’s divorce from regional- and individual-level variables: interaction effects *Source*: DHS (2003, 2008)

	Model 1a		Model 1b		Model 1c		Model 2a		Model 2b		Model 2c	
	Coef.	(SE)	Coef.	(SE)	Coef.	(SE)	Coef.	(SE)	Coef.	(SE)	Coef.	(SE)
Individual level^a^												
Education (none = ref.)												
Primary	0.276**	0.142	0.265*	0.142	0.260*	0.142	0.594***	0.212	0.416	0.286	0.669**	0.307
Secondary	0.658***	0.175	0.640***	0.175	0.639***	0.176	0.862***	0.253	1.032***	0.329	1.397***	0.355
Tertiary	0.840***	0.195	0.819***	0.195	0.823***	0.195	0.790***	0.285	0.254	0.444	0.583	0.404
Year of marriage (centred)	0.026*	0.015	0.031**	0.014	0.031**	0.014	0.031***	0.008	0.038***	0.006	0.038***	0.006
Regional level												
Crude divorce rate (time varying, *t* − 1)	0.150	0.122					0.554**	0.276				
Population density (rural = ref.)												
Intermediate			0.213	0.134					0.186	0.296		
Urban			0.380***	0.138					0.854***	0.329		
GDP per capita (regional %)					3.534***	1.454					7.915***	3.125
Interactions												
Primary*year of marriage (centred)	0.009	0.015	0.010	0.015	0.010	0.015						
Secondary*year of marriage (centred)	0.020	0.017	0.021	0.017	0.022	0.017						
Tertiary*year of marriage (centred)	−0.018	0.018	−0.016	0.018	−0.016	0.018						
Primary*CDR							−0.567**	0.281				
Secondary*CDR							−0.316	0.309				
Tertiary*CDR							−0.308	0.313				
Primary*rural (ref.)												
Primary*intermediate									−0.013	0.333		
Primary*urban									−0.533	0.361		
Secondary*rural (ref.)												
Secondary*intermediate									−0.120	0.377		
Secondary*urban									−0.864**	0.407		
Tertiary*rural (ref.)												
Tertiary*intermediate									0.558	0.485		
Tertiary*urban									0.113	0.496		
Primary*GDP per capita											−5.146	3.293
Secondary*GDP per capita											−8.235**	3.722
Tertiary*GDP per capita											−0.285	3.925
Intercept	−3.378***	0.555	−3.494***	0.559	−3.585***	0.564	−3.662***	0.567	−3.632***	0.592	−3.964***	0.609
Random intercept	0.130*	0.074	0.087	0.072	0.116*	0.061	0.129*	0.073	0.084	0.073	0.117*	0.061
Observations: *individual level*	205,910		205,994		205,994		205,910		205,994		205,994	
Observations: *regional level*	12		12		12		12		12		12	
Unexplained variance: *regional level*	0.004		0.002		0.003		0.004		0.002		0.003	
Total explained variance^b^	0.174		0.178		0.178		0.177		0.1835		0.1828	

^a^All models controlled for: marriage duration, age at marriage, age at marriage (squared), mother’s literacy, children (t − 1), children from other than spouse, age difference, mother tongue Kurdish, consanguinity, arranged marriage, childhood residence

^b^For details on the calculation of the total explained variance, see Snijders and Bosker (1999: 225)

* *p* < .05; ** *p* < .01; *** *p* < .001

Contrary to the Goode (1962) hypothesis, our findings show that the effect of education does not change over time for women with higher education, relative to women with no or primary education (Models 1). This means that *Hypothesis 5* is not confirmed. We find that the interaction term for primary education and regional CDR (Model 2a) is negative and significant. This means that the positive effect of education decreases for women with primary education in regions where divorce is more prevalent. We, however, do not find a significant effect for women with secondary or tertiary education, which implies that the effect of education decreases in regions where divorce is already more prevalent. The latter partly confirms our expectation (*Hypothesis 6*).

In Model 2b, we show that the probability of divorce for women with secondary education is lower in more densely populated regions. Similarly, in Model 2c we show that the risk of divorce for women with secondary education is lower in regions with higher average shares of GDP per capita. Both these findings correspond to our hypotheses (*Hypothesis 7* and *8*). It is likely that the costs of divorce are lower in more urbanized and wealthier regions, resulting in lower divorce risks for women with higher (secondary) education relative to the divorce risks of women with less (no) education (De Graaf and Kalmijn 2006; Goode 1962).

## Discussion

In this article, we examined regional patterns of divorce as well as the factors contributing to women’s probability of divorce in Turkey. Both the relationships between regional-level (macro) and individual-level (micro) characteristics were scrutinized. Our work adds to the existing literature on the risk of divorce in two important ways. First, the majority of studies that explain divorce risks focus on divorce within Western countries (mainly Europe and North America). Non-Western countries have received little attention thus far, even though demographic changes may alter family life substantially there too. Turkey is a particularly relevant case to study as it has witnessed major changes in family life events in recent years. Second, most studies have focused on individual characteristics in the study of divorce patterns. We add the societal regional context to our study for the case of Turkey where there is great regional variation in divorce risks. Our analyses point to three important findings.

First, since the rise of divorce in Turkey is relatively recent, divorce can be considered a “new family demographic behaviour” in the Turkish context. Theories on the diffusion of innovations postulate that new behaviours first emerge in large towns and cities amongst those with a higher socioeconomic status (Liefbroer and Doureleijn 2006; Nazio and Blossfeld 2002). Since we were interested in how the wider societal context of women affected their probability of divorce, we considered characteristics of the region they lived in. This regional context is particularly relevant for Turkey, given the huge variation in demographic behaviour and economic development between the 12 different regions. In line with our expectations, living in regions where the CDR is higher (*Hypothesis 1*), living in more urban regions (*Hypothesis 2*), and living in regions where the average shares of GDP per capita are higher (*Hypothesis 3*) increase the divorce risks of women. The fact that our study clearly shows the expected patterns for the influence of the context points to the relevance of including measures of societal environment in the study of demographic behaviour. Importantly, in addition to these regional measures, our findings also show that controlling for the composition of the population within these regions explains a large share of the between-region variation. The fact that these rather broad measures for the wider context already show such clear impact, indicates that social networks need to be studied in more detail to pinpoint the underlying mechanisms that lead to specific choices in the family life. This is relevant both in the case of Turkey as well as for societies in Europe and elsewhere.

We also found that both period and cohort effects drive women’s probability of divorce. Women married in more recent years are more likely to divorce, and the two time periods corresponding to two emancipating legislative changes also demonstrate an increased risk of divorce (*Hypotheses 4a* and *4b*). Additionally, we found that higher educated women have higher probabilities to divorce, as well as women that lived in urban regions or abroad during their childhood. While the information on the exact whereabouts of these women that were internationally mobile is limited, we could speculate that they are exposed to Western contexts where divorce was already more prevalent. This in turn increases their likelihood of divorce. Our data did not allow a more fine-grained analyses but future research should link exposure to different norms and value systems in order to better determine the effect of these experiences across the life course on individual life choices.

Second, we tested whether the relationship between women’s education and her probability to divorce will be more negative over time as divorce becomes more accepted (*Hypothesis 5*) (cf. Goode 1962). Our findings are to some extent supportive of this hypothesis, since the effect of education decreases over time. Nonetheless, women with a higher education remain more likely to divorce than their lesser-educated counterparts. This could be the result of the fact that the prevalence of divorce in Turkey is still relatively low, so that the costs of divorce remain relatively high, and those with a higher education remain the pioneers. We could speculate that when this trend of increasing divorce rates in Turkey continues, the effect of education will decrease or even become negative.

Third, we expected that the effect of women’s education on divorce would also be shaped by the regional context. In line with our expectations, the effect of women’s education becomes less positive in regions where divorce is more prevalent, more urban regions, and wealthier regions (*Hypothesis 6, 7, and 8)*. These findings could be explained by the fact that the costs of divorce decrease in regions with higher CDR, that are more urbanized and wealthier. As a result, the divorce risk of women with higher (mostly secondary) education decreases compared to the risk of divorce of women without education (De Graaf and Kalmijn 2006; Goode 1962).

This study suggests that for Turkey, modernization and the diffusion of new family norms are associated with in an increase of divorce. However, the cross-sectional nature of the DHS data limits the possibilities of studying in detail the time-varying effects of several relevant characteristics, such as education and employment. Future studies could carry this work further by studying sociodemographic processes in Turkey from a more pronounced life course perspective. Additionally, information about characteristics of the husband is limited in our data, and we were not able to distinguish between divorce and separation, whereby people live separated but have not officially divorced. This potentially underestimates our estimates of the probability of divorce in Turkey. Finally, collecting context variables over time for the 12 regions proved to be challenging. Future research could carry analyses on the role of divorce further by enriching the data with more detailed time-varying regional characteristics (for example, the labour market participation of women).

Notwithstanding these limitations, this study is amongst the few to consider the role of the regional context in shaping women’s divorce risk over time. Specifically, using multilevel discrete-time models, we were able to simultaneously estimate the effects of women’s individual characteristics as well as the regional characteristics on the probability of divorce over an extended period of time. This revealed the importance of period and cohort effects and the relevance of accounting for both individual *and* regional levels in order to better understand divorce patterns.

## Footnotes

1 Since we pool the two surveys, we de-normalized the weights using the following formula: *new weight* *=* *(DHS sample weight/1,000,000) * (female population aged 15–49 in survey year/sample size of survey year)*. Population numbers were derived from the 2000 and 2005 General Population Census (United Nations (UN) 2015). These new weights were added to the data file and used for descriptive analyses only.

2 Our analytical sample of divorced women included both those who did not as well as who did remarry although the latter category was very limited: in 2003, only 3.0% (*n* = 244) women and in 2008 only 2.5% (*n* = 186) remarried.

3 The original variable in the WVS (2007, V205) was coded 0 = never justifiable to 10 = always justifiable. We recoded this variable to a dichotomous variable with 0 = never justifiable (0–4) and 1 = justifiable (5>).
